# Effect of Hydralazine on Angiotensin II-Induced Abdominal Aortic Aneurysm in Apolipoprotein E-Deficient Mice

**DOI:** 10.3390/ijms242115955

**Published:** 2023-11-03

**Authors:** Yutang Wang, Owen Sargisson, Dinh Tam Nguyen, Ketura Parker, Stephan J. R. Pyke, Ahmed Alramahi, Liam Thihlum, Yan Fang, Morgan E. Wallace, Stuart P. Berzins, Ernesto Oqueli, Dianna J. Magliano, Jonathan Golledge

**Affiliations:** 1 Discipline of Life Science, Institute of Innovation, Science and Sustainability, Federation University Australia, Ballarat, VIC 3353, Australia; owensargisson@students.federation.edu.au (O.S.); ngutam372@gmail.com (D.T.N.); m.wallace@federation.edu.au (M.E.W.); s.berzins@federation.edu.au (S.P.B.); 2 Cardiology Department, Grampians Health Ballarat, Ballarat, VIC 3350, Australia; ernesto.oqueliflores@bhs.org.au; 3 School of Medicine, Faculty of Health, Deakin University, Geelong, VIC 3220, Australia; 4 Diabetes and Population Health, Baker Heart and Diabetes Institute, Melbourne, VIC 3004, Australia; 5 Queensland Research Centre for Peripheral Vascular Disease, College of Medicine and Dentistry, James Cook University, Townsville, QLD 4811, Australia; jonathan.golledge@jcu.edu.au; 6 Department of Vascular and Endovascular Surgery, The Townsville University Hospital, Townsville, QLD 4811, Australia

**Keywords:** abdominal aortic aneurysm, hydralazine, inflammation, atherosclerosis

## Abstract

The rupture of an abdominal aortic aneurysm (AAA) causes about 200,000 deaths worldwide each year. However, there are currently no effective drug therapies to prevent AAA formation or, when present, to decrease progression and rupture, highlighting an urgent need for more research in this field. Increased vascular inflammation and enhanced apoptosis of vascular smooth muscle cells (VSMCs) are implicated in AAA formation. Here, we investigated whether hydralazine, which has anti-inflammatory and anti-apoptotic properties, inhibited AAA formation and pathological hallmarks. In cultured VSMCs, hydralazine (100 μM) inhibited the increase in inflammatory gene expression and apoptosis induced by acrolein and hydrogen peroxide, two oxidants that may play a role in AAA pathogenesis. The anti-apoptotic effect of hydralazine was associated with a decrease in caspase 8 gene expression. In a mouse model of AAA induced by subcutaneous angiotensin II infusion (1 µg/kg body weight/min) for 28 days in apolipoprotein E-deficient mice, hydralazine treatment (24 mg/kg/day) significantly decreased AAA incidence from 80% to 20% and suprarenal aortic diameter by 32% from 2.26 mm to 1.53 mm. Hydralazine treatment also significantly increased the survival rate from 60% to 100%. In conclusion, hydralazine inhibited AAA formation and rupture in a mouse model, which was associated with its anti-inflammatory and anti-apoptotic properties.

## 1. Introduction

Abdominal aortic aneurysm (AAA) rupture is estimated to be responsible for approximately 200,000 deaths worldwide each year [1]. Open surgical and endovascular aortic repair are the only current treatments for AAA, but they are associated with safety and durability problems [2]. The perioperative mortality rate of open surgical AAA repair is approximately 5% [3,4], and for endovascular AAA repair, it is about 2% [2]. Up to 20% of patients need re-intervention after endovascular AAA repair [2]. AAA repair is only recommended for women with an AAA diameter of ≥50 mm and men with an AAA diameter of ≥55 mm, as randomized controlled trials suggest repair of small aneurysms does not reduce mortality [2,5]. Patients with small AAAs are managed conservatively using repeated imaging to monitor the size of the AAA. Up to 70% of patients undergoing imaging surveillance eventually require AAA repair [2,5]. Medications are needed to limit AAA growth, but previously tested drugs have been ineffective in randomized clinical trials [6,7].

Aortic inflammation and loss of vascular smooth muscle cells (VSMCs) are two hallmark features of AAA pathology [2]. Hydralazine, an antihypertensive medication, has been shown to inhibit inflammation [8,9] in various disease models (spinal cord injury [10], kidney injury [11], and sepsis [12]), and also limit apoptosis of cardiomyocytes [13]. Whether hydralazine inhibits the apoptosis of VSMCs is unknown. In this study, we aimed to investigate whether hydralazine inhibits apoptosis of VSMCs in vitro and whether it inhibits AAA formation in vivo.

To investigate whether hydralazine inhibits the apoptosis of VSMCs in vitro, we used two apoptosis inducers, i.e., acrolein and hydrogen peroxide (H_2_O_2_). The oxidant acrolein is considered one of the most toxic and harmful components of cigarette smoke [14,15], the latter being a major risk factor for AAA formation [2]. The oxidant H_2_O_2_ is thought to play an important role in AAA pathogenesis [16].

To investigate whether hydralazine inhibits AAA formation in vivo, we used a mouse model in which AAA was induced by a subcutaneous infusion of angiotensin II. We compared the AAA incidence, size, and survival between two groups of mice treated with or without hydralazine. As this angiotensin II infusion-induced AAA model is also a model of atherosclerosis [17] and cardiac hypertrophy [18,19], we simultaneously investigated whether hydralazine affected these two conditions.

## 2. Results

### 2.1. Hydralazine Decreased Inflammatory Cytokine Expression in Cultured VSMCs

**Figure 1:**
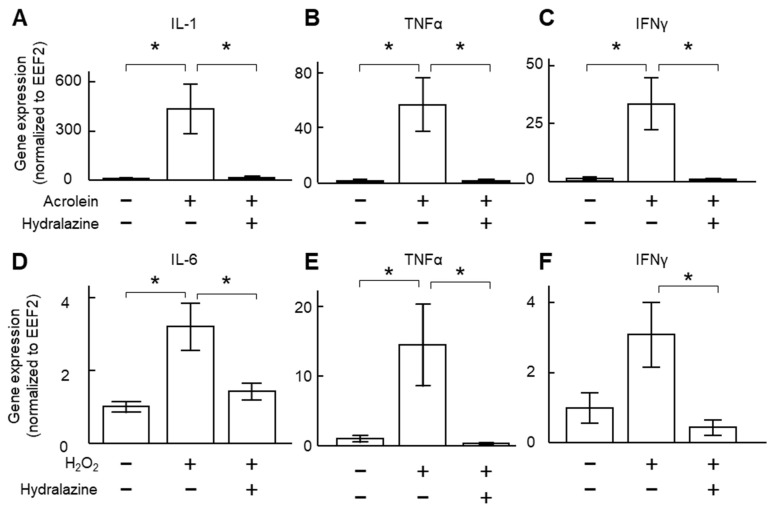
Effect of hydralazine on inflammatory gene expression. (**A**–**C**) VSMCs were incubated with acrolein (2 μg/mL) in the presence (+) or absence (−) of hydralazine (100 μM) for 24 h. mRNA was then extracted, and gene expression of IL-1 (**A**), TNFα (**B**), and IFNγ (**C**) was analyzed using qPCR. (**D**–**F**), VSMCs were treated with hydrogen peroxide (H_2_O_2_, 800 µM) in the presence or absence of hydralazine (100 μM) for 24 h. mRNA was then extracted, and gene expression of IL-6 (**D**), TNFα (**E**), and IFNγ (**F**) was analyzed using qPCR. *n*= 5–6. Error bars = standard error. Data were analyzed using a Kruskal–Wallis one-way ANOVA. * *p* < 0.05. EEF2, eukaryotic translation elongation factor 2; IFNγ, interferon-gamma; IL, interleukin; TNFα, tumor necrosis factor-alpha; VSMCs, vascular smooth muscle cells.

We used two separate oxidants (acrolein and H_2_O_2_) to induce cytokine expression in VSMCs. Incubation of the cells with either of these two compounds increased the expression of inflammatory cytokines (Figure 1). Co-incubation of the cells with hydralazine (100 μM) decreased gene expression of interleukin-1 (IL-1), IL-6, tumor necrosis factor-alpha (TNFα), and interferon-gamma (IFNγ, Figure 1). Enzyme-linked immunosorbent assay (ELISA) results confirmed that H_2_O_2_ increased IL-6 protein levels, and hydralazine (100 μM) mitigated such an increase (Figure S1).

### 2.2. Hydralazine Decreased Acrolein- and H_2_O_2_-Induced Cell Death in Cultured VSMCs 

**Figure 2:**
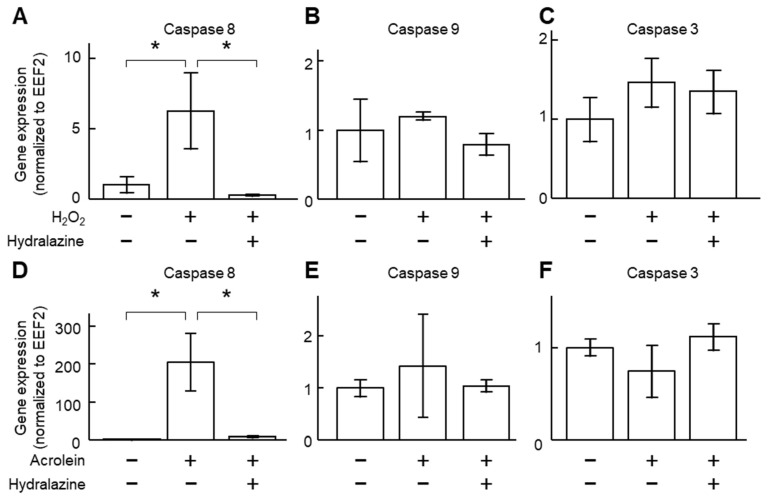
Effect of hydralazine on caspase gene expression. VSMCs were treated with acrolein (2 μg/mL, (**A**–**C**)) or hydrogen peroxide (800 µM, (**D**–**F**)) in the presence (+) or absence (−) of hydralazine (100 μM) for 24 h. mRNA was then extracted, and gene expression of caspase 8 (**A**,**D**), caspase 9 (**B**,**E**), and caspase 3 (**C**,**F**) was analyzed using qPCR. *n* = 5–6. Error bars = standard error. Data were analyzed using a Kruskal–Wallis one-way ANOVA. * *p* < 0.05. EEF2, eukaryotic translation elongation factor 2; H_2_O_2_, hydrogen peroxide; VSMCs, vascular smooth muscle cells.

**Figure 3:**
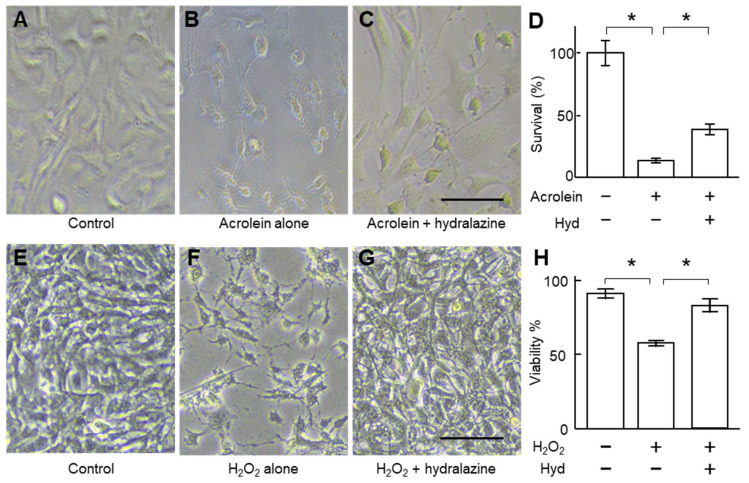
Effect of hydralazine on VSMC survival. (**A**–**D**), VSMCs were incubated with acrolein (4 μg/mL) in the presence (+) or absence (−) of hydralazine (100 μM) for 24 h. Representative cell images of control cells (**A**) and cells incubated with acrolein in the absence (**B**) or presence of hydralazine (**C**). Scale bar = 50 μm. (**D**) The survival of cells, assessed by the MTS assay; *n* = 8. (**E**–**H**), VSMCs were incubated with hydrogen peroxide (H_2_O_2_) in the presence (+) or absence (−) of hydralazine (100 μM) for 24 h. Representative cell images of control cells (**E**) and cells treated with H_2_O_2_ (800 µM) in the absence (**F**) or presence of hydralazine (**G**). (**H**) The cell viability, assessed by the trypan blue assay; *n* = 4. Error bars = standard error. Data were analyzed using a Kruskal–Wallis one-way ANOVA. * *p* < 0.05. VSMCs, vascular smooth muscle cells.

**Figure 4:**
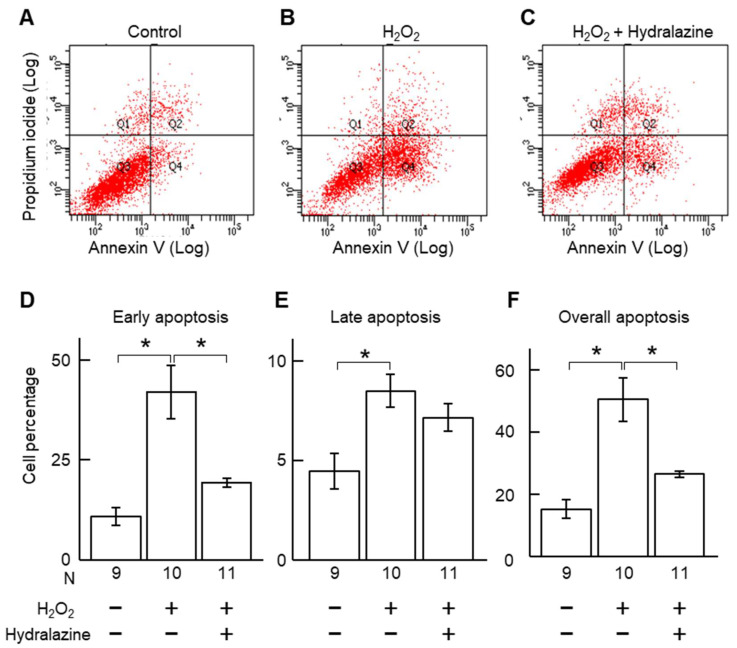
Effect of hydralazine on VSMC apoptosis induced by H_2_O_2_. (**A**–**C**), Flow cytometry graphs of control VSMC cells (**A**), and cells treated with H_2_O_2_ (100 μM, **B**) or co-treated with H_2_O_2_ (100 μM) and hydralazine (100 μM, **C**). Cells were harvested and then stained with propidium iodide and annexin V. (**D**–**F**), Percentage of apoptotic cells. Cells in the early apoptotic stage (**A**) were defined as annexin V-high and propidium iodide-low. Cells in the late apoptotic stage (**E**) were defined as annexin V-high and propidium iodide-high. Overall apoptotic cells (**F**) were defined as annexin V-high. Error bars = standard error. Data were analyzed using a Kruskal–Wallis one-way ANOVA. * *p* < 0.05. H_2_O_2_, hydrogen peroxide; VSMCs, vascular smooth muscle cells.

Acrolein and H_2_O_2_ are two commonly used apoptosis inducers [20,21] and may play a role in AAA pathogenesis [14,15,16]. Incubation of VSMCs with acrolein and H_2_O_2_ increased caspase 8 gene expression and did not affect caspases 3 and 9 (Figure 2), suggesting that these two compounds activated the extrinsic apoptotic pathway. Co-incubation of the cells with hydralazine (100 μM) mitigated the increase in caspase 8 gene expression, suggesting that hydralazine may inhibit apoptosis induced by acrolein and H_2_O_2_. Indeed, hydralazine attenuated cell death induced by acrolein and H_2_O_2_ (Figure 3). Flow cytometry results confirmed that hydralazine (100 μM) decreased the percentage of cells undergoing apoptosis induced by H_2_O_2_, as assessed by annexin V staining (Figure 4).

### 2.3. Hydralazine Treatment Protected Apolipoprotein E-Deficient (ApoE^−/−^) Mice against AAA

**Figure 5:**
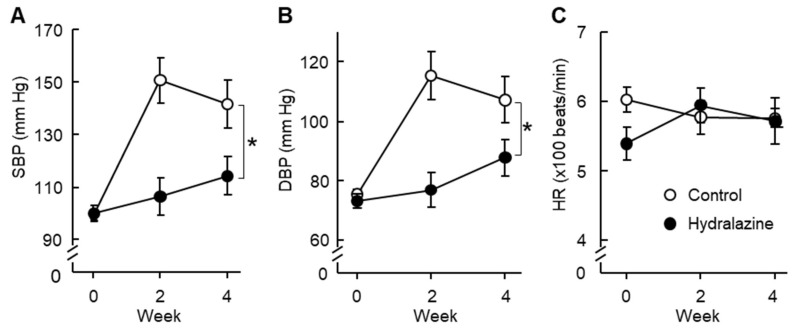
Effect of hydralazine treatment on blood pressure and heart rate. The basal (Week 0) systolic blood pressure (SBP, (**A**)), diastolic blood pressure (DBP, (**B**)), and heart rate (HR, (**C**)) of the mice were measured by the tail-cuff method. Then, the mice in the hydralazine-treated group were treated with hydralazine (24 mg/kg/day, filled circles), and the mice in the control group received vehicle (water, open circles) until the end of the experiment. 3 days after the initiation of the hydralazine treatment, angiotensin II (1 µg/kg body weight/min) was subcutaneously administered to all the mice for 28 days. Blood pressure and heart rate were measured again at 2 and 4 weeks after angiotensin II administration. Error bars = standard error. * *p* < 0.05. Hydralazine group, *n* = 10. Control groups: Week 0, *n* = 10; week 2, *n* = 6; and week 4, *n* = 6.

Angiotensin II infusion increased blood pressure in ApoE^−/−^ mice, and hydralazine treatment attenuated this increase (Figure 5A,B). Angiotensin II or hydralazine did not affect heart rate (Figure 5C).

**Figure 6:**
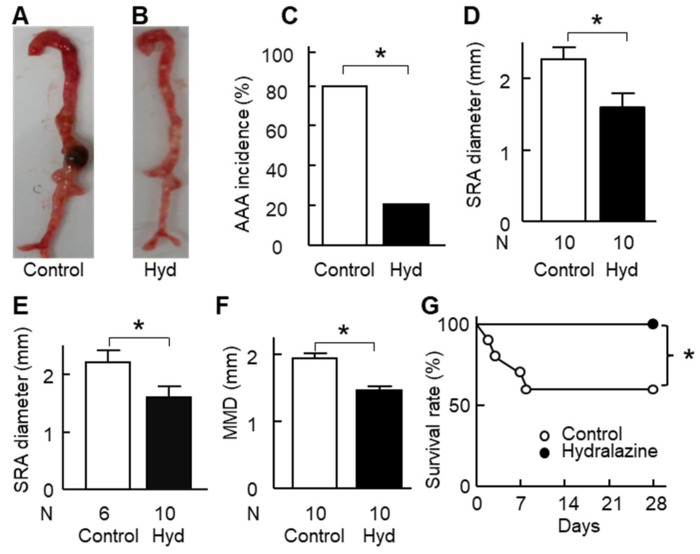
Effect of hydralazine treatment on abdominal aortic aneurysm (AAA). Two groups of mice were used: the control group and the hydralazine-treated group (Hyd). Mice in the latter group were treated with hydralazine (24 mg/kg/day) and mice in the former group were treated with vehicle (water) until the end of the experiment. 3 days after the initiation of the hydralazine treatment, angiotensin II (1 µg/kg body weight/min) was subcutaneously administered to all the mice for 28 days to induce AAA. (**A**,**B**), Representative images of aortas of mice in the absence (**A**) or presence of hydralazine (**B**). (**C**), AAA incidence of the mice. (**D**,**E**), Diameter of the suprarenal aorta (SRA) of the mice. (**D**) panel included all the mice (surviving mice plus dead mice due to aortic rupture) and the (**E**) panel included surviving mice only. (**F**), Mean maximum aortic diameter (MMD) of the mice which was the mean of the maximum diameter of the following four aortic segments: aortic arch, thoracic aorta, suprarenal aorta, and infrarenal aorta. (**G**), Kaplan–Meier survival curve. Error bars = standard error. * *p* < 0.05.

Angiotensin II infusion induced AAA formation in ApoE^−/−^ mice (Figure 6A,B, Figure S2). Hydralazine treatment decreased the incidence of AAA from 80% to 20% (*p* < 0.05, Figure 6C) and suprarenal aortic diameter by 32%, from 2.26 mm to 1.53 mm (*p* < 0.05, Figure 6D). When the four animals that died of aortic rupture were excluded, similar results were obtained (Figure 6E and Figure S3). Hydralazine treatment also decreased aortic arch diameter, thoracic aorta diameter, and mean maximum aortic diameter (*p* < 0.05, Figure S3 and Figure 6F). In addition, hydralazine treatment protected the mice against aortic rupture and enhanced the survival rate from 60% to 100% (*p* < 0.05, Figure 6G). Consistent with the in vitro results, in vivo data showed that hydralazine treatment decreased apoptosis in mouse suprarenal aortas (Figure S4).

### 2.4. Hydralazine Treatment Protected Mice against Atherosclerosis and Cardiac Hypertrophy 

**Figure 7:**
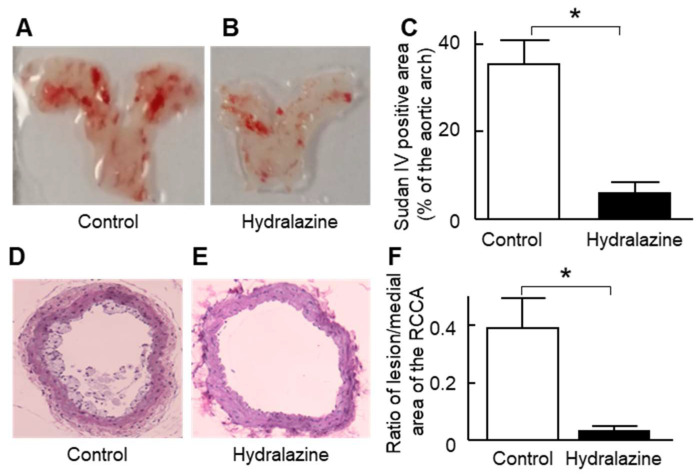
Effect of hydralazine treatment on atherosclerosis. Mice, treated with or without hydralazine (24 mg/kg/day), were sacrificed at the end of the 28-day subcutaneous infusion of angiotensin II (1 μg/kg body/min) for atherosclerosis assessment. (**A**,**B**) Representative images of Sudan IV staining of the aortic arch in the control (**A**) and hydralazine-treated mice (**B**). (**C**) The ratio of the Sudan IV positive area over the entire arch surface area. (**D**,**E**) The representative image of H&E staining of the right common carotid artery (RCCA) in the control (**D**) and hydralazine-treated mice (**E**). (**F**), The ratio of the lesion area over the medial area. *n* = 6 in the control group and *n* = 10 in the hydralazine group. Error bars = standard error. Image magnification = 10×. Data were analyzed using the Mann–Whitney U test. * *p* < 0.05. H&E: hematoxylin and eosin.

As this AAA model is also a model of atherosclerosis [17] and cardiac hypertrophy [18,19], we also investigated whether hydralazine affected those two conditions in this animal model. The results showed that hydralazine treatment significantly reduced atherosclerosis at the aortic arch and right common carotid artery (Figure 7), heart weight, and cardiomyocyte width (Figure S5).

## 3. Discussion

This study found that incubation of VSMCs with hydralazine in vitro inhibited the expression of pro-inflammatory genes and reduced apoptosis. Hydralazine also inhibited AAA formation and rupture in ApoE^−/−^ mice. To our knowledge, this is the first report suggesting that hydralazine decreases AAA formation. Our results are consistent with previous reports on cerebral aneurysms. In one study, hydralazine administration decreased the incidence of advanced-stage cerebral aneurysms from 50% to 9% in a rat model [22]. In another study, hydralazine protected against cerebral aneurysmal rupture in a mouse model [23]. 

In contrast, our results are different from those reported by Cassis and colleagues [24], who found that hydralazine did not affect angiotensin II-induced AAA formation in ApoE^−/−^ mice. The reason for this disparity is unclear, but may relate to differences in study designs, reporting, and methods [24]. In keeping with many previous reports, approximately 40% of mice had an aortic rupture in the current study [25,26]. In the investigation by Cassis and colleagues, aortic rupture was not reported, and it is possible such mice were excluded. Drinking water containing hydralazine was freshly prepared three times a week in the current study, and the preparation of hydralazine-containing drinking water was not reported in the prior study [24]. Personal communication with the authors confirmed that they had freshly prepared hydralazine every three days, which was slightly less frequent than in the current study. Whether this slight difference in hydralazine preparation contributed to the discrepancy observed is unknown. The systolic blood pressure of the hydralazine-treated mice in the previous study was 135 mm Hg [24], compared to 114 mm Hg in the current study, suggesting that hydralazine was having a more powerful effect in our investigation, where there was more frequent replenishment of hydralazine in drinking water. This suggests that differences in hydralazine preparation might have contributed to the disparity in findings.

Aortic inflammation and VSMC apoptosis are key features of AAA pathology [2]. Inhibition of inflammation, e.g., by blocking IL-6 [27] and C-X-C motif chemokine receptor 2 (CXCR2) [28], inhibits AAA formation in mouse models. In addition, interventions that inhibit VSMC apoptosis, such as 2-hydroxypropyl-β-cyclodextrin [29] and mitochondrial fission inhibitors [30], have been shown to attenuate AAA development in mouse models. Our results showed that hydralazine inhibited expression of some pro-inflammatory genes and apoptosis of VSMCs, suggesting that the anti-AAA effect of hydralazine may be mediated, at least in part, by its anti-inflammatory and anti-apoptotic properties.

Whether lowering blood pressure contributed to the anti-AAA effect of hydralazine is unknown. It has been shown that hypertension is an important risk factor for AAA in humans [31]. Increasing blood pressure alone does not result in AAA formation in animal studies [24], and antihypertensive drugs such as beta-blockers do not inhibit AAA progression in humans [32]. Therefore, lowering blood pressure may not be the key mechanism underlying the anti-AAA effect of hydralazine reported in this study. 

Hydralazine did not increase the heart rate of mice with hypertension induced by angiotensin II in the present study. This is consistent with previous reports that hydralazine did not affect heart rate in mice with hypertension induced by angiotensin II [33] or in genetically hypertensive mice [34]. However, it has been reported that hydralazine increases heart rate in normotensive mice [35,36]. The reason underlying this inconsistency is unclear. One might speculate that hydralazine decreases the blood pressure of normotensive mice to a sub-normal range, and this may compromise blood supply to vital organs. Consequently, mice may increase their heart rate to increase cardiac output and maintain sufficient blood supply to vital organs. However, in hypertensive mice, hydralazine may decrease blood pressure to a normal range; in this case, blood supply may not be compromised, and thus the mice may not respond with an increase in heart rate.

We also observed that hydralazine has an anti-atherosclerotic effect in ApoE^−/−^ mice. Our results are consistent with some previous reports. Hydralazine has been shown to decrease atherosclerosis induced by deoxycorticosterone acetate salt and high-fat diet administration [37]. In addition, hydralazine decreased atherosclerosis in ApoE^−/−^ mice fed a normal diet [38]. The anti-atherosclerotic effect of hydralazine may be due to its blood pressure-lowering and anti-inflammatory properties, as hypertension and inflammation are key treatment targets for limiting atherosclerosis-associated cardiovascular events [39,40,41]. Nonetheless, some reports have suggested that hydralazine does not decrease atherosclerosis. For example, one study showed that hydralazine did not affect atherosclerosis in the aortic root of ApoE^−/−^ mice fed a high-fat diet [42], although the authors of this study did not investigate whether hydralazine affects atherosclerosis in other vascular territories. Other investigators [35,43] have reported that hydralazine does not significantly reduce the severity of aortic atherosclerosis in comparison to controls.

Angiotensin II infusion also induces cardiac hypertrophy in mice [18,19]. The current study suggests that hydralazine protects mice against cardiac hypertrophy, as indicated by lighter heart weights and smaller widths of cardiomyocytes. Our results are consistent with the report from Vial and colleagues [44], who showed that hydralazine protected against cardiac hypertrophy in Wistar rats treated with deoxycorticosterone acetate salt. This protective effect of hydralazine may result from its blood pressure-lowering and anti-inflammatory properties. Hypertension is a major risk factor for cardiac hypertrophy [45], and lowering blood pressure with antihypertensive drugs reduces cardiac hypertrophy in humans [46]. It is known that cardiac inflammation is increased in cardiac hypertrophy, and inhibition of inflammation could decrease cardiomyocyte enlargement [47]. 

In conclusion, our study showed that hydralazine inhibited AAA formation in ApoE^−/−^ mice infused with angiotensin II, which may be mediated by its anti-inflammatory and anti-apoptotic effects. A limitation of this study is its sample size. Whether hydralazine inhibits AAA in other animal models or in humans needs to be investigated in the future. In addition, in this angiotensin II infusion mouse model, hydralazine treatment protected the mice from developing atherosclerosis and cardiac hypertrophy. The underlying mechanisms by which hydralazine exerts its effects need to be further investigated in the future.

## 4. Methods

### 4.1. Animals

Twenty male ApoE^−/−^ mice were purchased from the Animal Resources Centre, Perth, Australia. All experiments were conducted in a temperature-controlled animal house (21 ± 1 °C) under a 12:12 h light–dark cycle, and mice were given a normal diet and water ad libitum.

### 4.2. Experimental Protocol

The mice (14 weeks old) were randomized into a control group and a hydralazine-treated group (*n* = 10 per group). The mice in the former group received plain drinking water, whereas those in the latter group received hydralazine via drinking water (24 mg/kg/day) throughout the experiment [48]. The hydralazine-containing drinking water was freshly prepared three times a week (Mondays, Wednesdays, and Fridays). Three days after the initiation of the hydralazine treatment, angiotensin II was administered subcutaneously to all the mice via a micro-osmotic pump (Model 2004, ALZET, DURECT Corporation, Cupertino, CA, USA) at a rate of 1 µg/kg body weight/min for 28 days to induce AAA [26]. Blood pressure and heart rate were measured in conscious mice at 0 weeks (before hydralazine treatment), 2 weeks, and 4 weeks after angiotensin II infusion using the tail-cuff method [49].

### 4.3. Morphometry Measurement of Aortic Diameter

Morphometry measurements were performed on aortas harvested from the surviving mice following euthanasia at the end of the experiment or at the autopsy of the mice who died of aortic rupture during the experiment, as previously described [26]. In brief, the harvested aortas were digitally photographed together with a ruler. Maximum external diameters of the aortic arch, thoracic aorta, suprarenal aorta, and infrarenal aorta were determined from the images using computer-aided analysis (Adobe Photoshop, version 24.6, Adobe Systems Incorporated, San Jose, CA, USA). The mean maximum aortic diameter was calculated as the mean of the maximum diameter of the aortic arch, thoracic aorta, suprarenal aorta, and infrarenal aorta. This morphometrical method has been shown to be highly reproducible [26].

### 4.4. Quantification of the Atherosclerotic Lesion Area

Atherosclerosis in the aortic arch was quantified by en face Sudan IV staining of the aortic arch as described previously [17] and by morphometry analysis of the right common carotid artery after hematoxylin and eosin (H&E) staining as described previously [17]. 

### 4.5. Cell Width of Cardiomyocytes

Five hearts from control mice and four hearts from hydralazine-treated mice were randomly selected. The left ventricles of the mice were formalin-fixed and paraffin-embedded before sectioning (5 μm thick). The sections were stained with H&E, and images were captured using a Nikon Eclipse 80i microscope (Nikon, Tokyo, Japan) with a CCD (charge-coupled device) camera at ×20 magnification. Twenty cardiomyocytes were randomly selected from each image, and their widths were measured 10 times using Adobe Photoshop. The mean width of cardiomyocytes was then calculated.

### 4.6. Cell Culture

VSMCs were isolated from the mouse aorta [50] and cultured as previously described [17]. In brief, VSMCs were cultured at 37 °C in Dulbecco’s modified Eagle medium (DMEM) supplemented with 10% *v*/*v* fetal bovine serum, 100 U/mL penicillin, and 100 μg/mL streptomycin in a standard cell culture incubator.

### 4.7. MTS Assay

VSMCs were cultured in 96-well plates (2 × 10^4^ cells/well) for 24 h before the addition of acrolein (4 μg/mL) with or without hydralazine (100 μM) [51,52] for another 24 h. Cell numbers were then assessed using an MTS assay kit (Abcam, Cambridge, UK) as previously described [50]. 

### 4.8. Trypan Blue Assay

The trypan blue assay was conducted as previously described [53]. In brief, 2 mL of VSMCs (5 × 10^5^ cells/mL) were placed in wells of 6-well plates and incubated in an incubator at 37 °C for 24 h. Then, the cells were incubated with H_2_O_2_ (800 μM) with or without hydralazine (100 µM). After another 24 h, the cells were trypsinized, stained with trypan blue, and the viability of the cells was determined using the Countess Automated Cell Counter (Invitrogen, Waltham, MA, USA).

### 4.9. Gene Expression Analysis

Effect of hydralazine on inflammatory and apoptotic gene expression in VSMCs treated with acrolein: VSMCs (1 × 10^6^ cells per well) were cultured in 3 wells of 6-well plates; Well 1 served as a control; Well 2 was incubated with acrolein alone (2 μg/mL); and Well 3 was co-incubated with acrolein (2 μg/mL) and hydralazine (100 μM). RNA was then extracted using the TRI reagent (Merck).

Effect of hydralazine on inflammatory and apoptotic gene expression in VSMCs treated with H_2_O_2_: the cells were treated as described in the previous paragraph, except that the acrolein was replaced with H_2_O_2_ (800 μM).

The extracted RNA was reverse transcribed to cDNA using the High-Capacity Reverse Transcription Kit (Life Technologies, Carlsbad, CA, USA). Gene expression was assessed by quantitative PCR using SYBR reagents (Bioline Global Pty Ltd, Gregory Hills, Australia). Primer sets were outlined in Table S1. The cycling conditions were 40 cycles of 95 °C for 15 s, 58 °C for 20 s, and 72 °C for 20 s. Relative gene expression was assessed using the 2^−ΔΔCt^ method [54]. Eukaryotic translation elongation factor 2 (EEF2) served as a housekeeping gene for normalizing gene expression [55].

### 4.10. Enzyme-Linked Immunosorbent Assay (ELISA) 

VSMCs (5 × 10^5^ cells/well) were placed in wells of 6-well plates and incubated in an incubator at 37 °C. After 24 h, the DMEM supplemented with 10% fetal bovine serum in the wells was replaced with 2 mL of fresh DMEM supplemented with 1% fetal bovine serum. The cells were then incubated with H_2_O_2_ (200 μM) with or without hydralazine (100 µM). After another 24 h, the culture medium was collected, and interleukin 6 protein levels in the culture medium were quantified using an enzyme-linked immunosorbent assay kit (Sigma-Aldrich Pty Ltd, Bayswater, Australia) according to the manufacturer’s instructions.

### 4.11. Flow Cytometry Assay 

VSMCs (5 × 10^5^ cells/well) were placed in wells of 6-well plates and were incubated with H_2_O_2_ (200 μM) with or without hydralazine (100 µM). After 24 h, the nonadherent cells in the culture medium were collected, and the adherent cells were trypsinized. Total cells (both adherent and nonadherent) were then used to determine apoptosis using BD Pharmingen reagents: Annexin V– BV421, Propidium Iodide (PI), and Annexin binding buffer, according to the manufacturer’s instructions [56]. Briefly, cells were washed and resuspended in binding buffer at a concentration of 2 × 10^6^ cells per ml, before being filtered and stained with annexin V and propidium iodide. Cell numbers were counted using the Becton-Dickson LSRFortessa X-20 flow cytometer (Franklin Lakes, NJ, USA) and analyzed using the BD FACSDiva software (v9.0). Apoptotic states were defined as previously described [57]: early apoptotic cells were defined as annexin V-high and propidium iodide-low; late apoptotic cells were defined as annexin V-high and propidium iodide-high; and overall apoptotic cells were defined as annexin V-high.

### 4.12. Terminal Deoxynucleotidyl Transferase Mediated dUTP Nick-End Labeling (TUNEL) Assay

Cell apoptosis was assessed by the TUNEL kit (Abcam) according to the manufacturer’s instructions as previously described [58]. In brief, 5 mice from each group were randomly chosen, and 5 mm thick paraffin-embedded suprarenal aortic sections were dewaxed and treated with proteinase K. After endogenous peroxidase was blocked with 3% H_2_O_2_, apoptotic cells were labeled with TdT Enzyme and detected using a 3,30-diaminobenzidine (DAB) substrate. The sections were then counterstained with methyl green. Four high magnification fields (×40) were randomly chosen from each sample, and TUNEL-positive cells were counted. The mean of the positive cells was calculated to represent cell apoptosis for each sample.

### 4.13. Statistical Analyses

The difference between two groups was analyzed using the Mann–Whitney U-test [59], and the difference among three groups was analyzed using a Kruskal–Wallis one-way ANOVA. The difference in blood pressure and heart rate between two groups (with or without hydralazine) was analyzed using multiple linear regression: dependent variable = blood pressure or heart rate, and independent variables = groups (with or without hydralazine) and time. Kaplan–Meier survival curves were analyzed using the log-rank (Mantel-Cox) test. The difference in AAA incidence was analyzed using Fisher’s exact test [59,60]. All tests were two-sided, and a *p* value of <0.05 was regarded as statistically significant. All analyses were performed using SPSS version 27.0 (IBM SPSS Statistics for Windows, Armonk, NY, USA, IBM Corporation).

## Data Availability

Not applicable.

## Supplementary Materials

The following supporting information can be downloaded at: https://www.mdpi.com/article/10.3390/ijms242115955/s1.
