# Supplementary material for: Effect of Hydralazine on Angiotensin II-Induced Abdominal Aortic Aneurysm in Apolipoprotein E-Deficient Mice
Source: Int J Mol Sci. 2023 Nov 3;24(21):15955. doi: 10.3390/ijms242115955 (PMC10650676; doi:10.3390/ijms242115955)
Supplement: Supplementary file 1 [file ijms-24-15955-s001.zip › ijms-2544256-supplementary.pdf]

International Journal of *Molecular Sciences*

MDPI

*Supplementary figures and tables*

# Effect of hydralazine on angiotensin II-induced abdominal aortic aneurysm in apolipoprotein E-deficient mice

**Yutang Wang [1,*,†], Owen Sargisson [1,†], Dinh Tam Nguyen [1], Ketura Parker [1], Stephan J.R. Pyke [1], Ahmed Alramahi [1], Liam Thihlum [1], Yan Fang [1], Morgan E. Wallace [1], Stuart P. Berzins [1], Ernesto Oqueli [2,3], Dianna J. Magliano [4,*], Jonathan Golledge [5,6]**

1 Discipline of Life Science, Institute of Innovation, Science and Sustainability, Federation University Australia, Ballarat, VIC, Australia.
2 Cardiology Department, Grampians Health Ballarat, Ballarat, VIC, Australia
3 School of Medicine, Faculty of Health, Deakin University, Geelong, VIC, Australia
4 Diabetes and Population Health, Baker Heart and Diabetes Institute, Melbourne, VIC, Australia
5 Queensland Research Centre for Peripheral Vascular Disease, College of Medicine and Dentistry, James Cook University, Townsville, QLD, Australia
6 Department of Vascular and Endovascular Surgery, The Townsville University Hospital, Townsville, QLD, Australia

* Correspondence: yutang.wang@federation.edu.au or Dianna.Magliano@baker.edu.au
† These authors contributed equally to this work.

**Table S1**: List of mouse primer sequences.

| Name | Forward primer | Reverse primer |
|---|---|---|
| Caspase 3 | 5'-CGTGGTTCATCCAGTCCCTTT-3' | 5'-ATTCCGTTGCCACCTTCCT-3' |
| Caspase 8 | 5'-TCTACCCTCCAGCTCTCTGT-3' | 5'-AGAGGTAGAAGAGCTGTAACCTTAT-3' |
| Caspase 9 | 5'-ACCTTCCCAGGTTGCCAATG-3' | 5'-CCCCGAGCCTCATGAAGTTTT-3' |
| EEF2 | 5'-ACATGTCAGTGATCGCCCAT-3' | 5'-GAGATGGTGGTGGATTTGATTG-3' |
| IFNγ | 5'-TGGCTGTTTCTGGCTGTTACT-3' | 5'-TCATGTCACCATCCTTTTGCCA-3' |
| IL-1 | 5'-GCCACCTTTTGACAGTGATGAA-3' | 5'-GACAGCCCAGGTCAAAGGTT-3' |
| IL-6 | 5'-CGGCCTTCCCTACTTCACAA-3' | 5'-GCCATTGCACAACTCTTTCTCA-3' |
| TNF-α | 5'-TAGCCCACGTCGTAGCAAAC-3' | 5'-ACAAGGTACAACCCATCGGC-3' |

Abbreviations: EEF2, eukaryotic translation elongation factor 2; IFNγ, interferon-gamma; IL, interleukin; T*NF-α,* tumor necrosis factor-alpha.

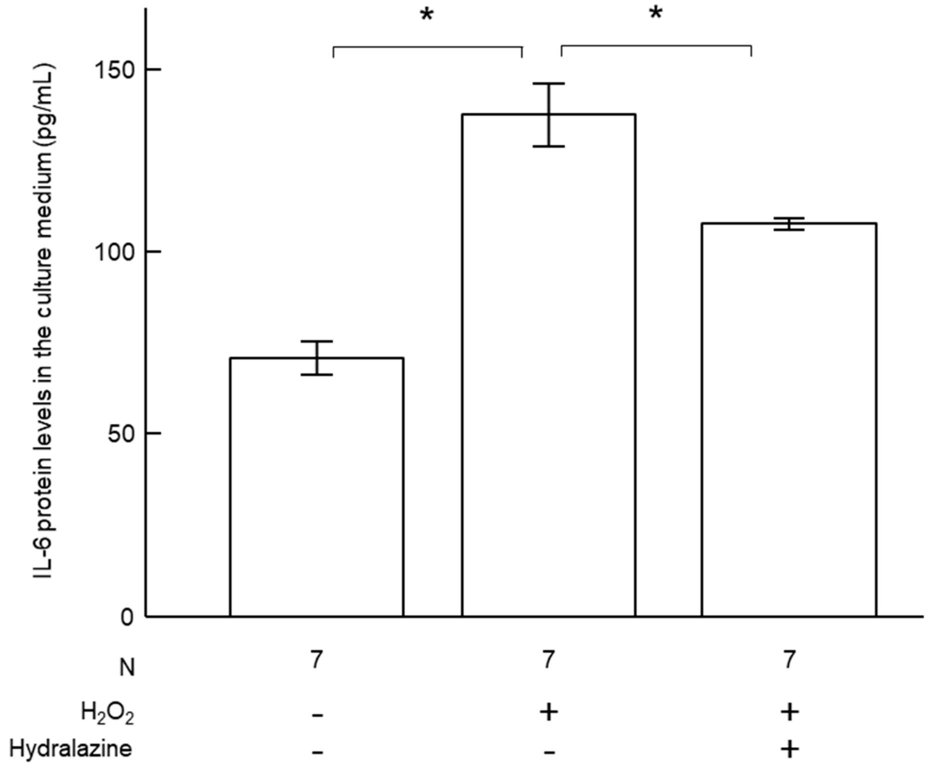


**Figure S1**: Effect of hydralazine on IL-6 protein levels in culture media. VSMCs were treated with $H_2O_2$ (200 μM) or co-treated with $H_2O_2$ and hydralazine (100 μM) for 24 h. IL-6 protein in the culture media was then quantified using ELISA. Error bars = standard error. Data were analyzed using Kruscal Wallis one-way ANOVA. * $P<0.05$. ELISA, enzyme-linked immunosorbent assay; $H_2O_2$, hydrogen peroxide; IL-6, interleukin 6; VSMCs, vascular smooth muscle cells.

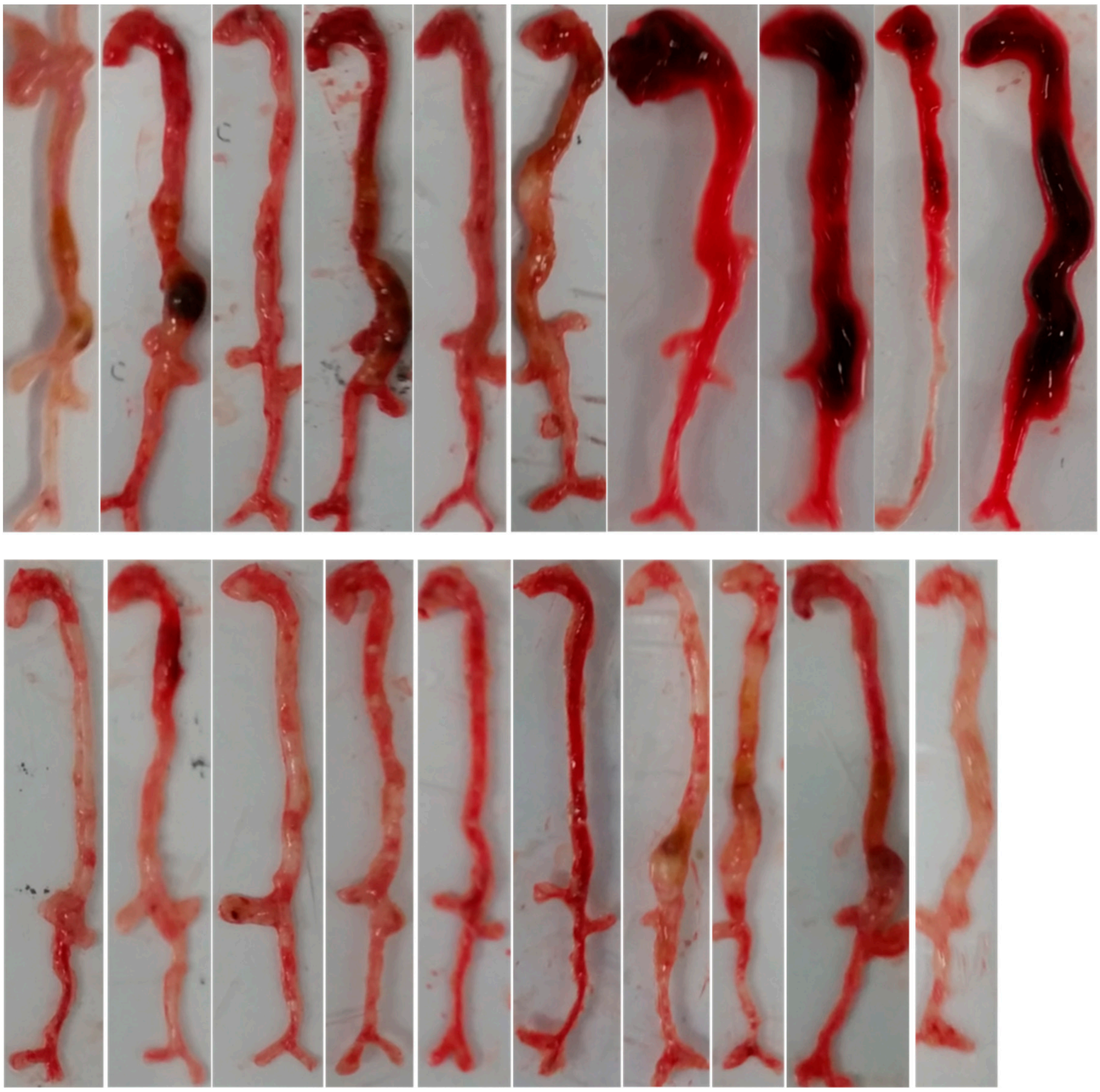

**Figure S2**: Aortic images. The top row images were the aortas of the control mice and the bottom row images were the aortas of the mice treated with hydralazine.

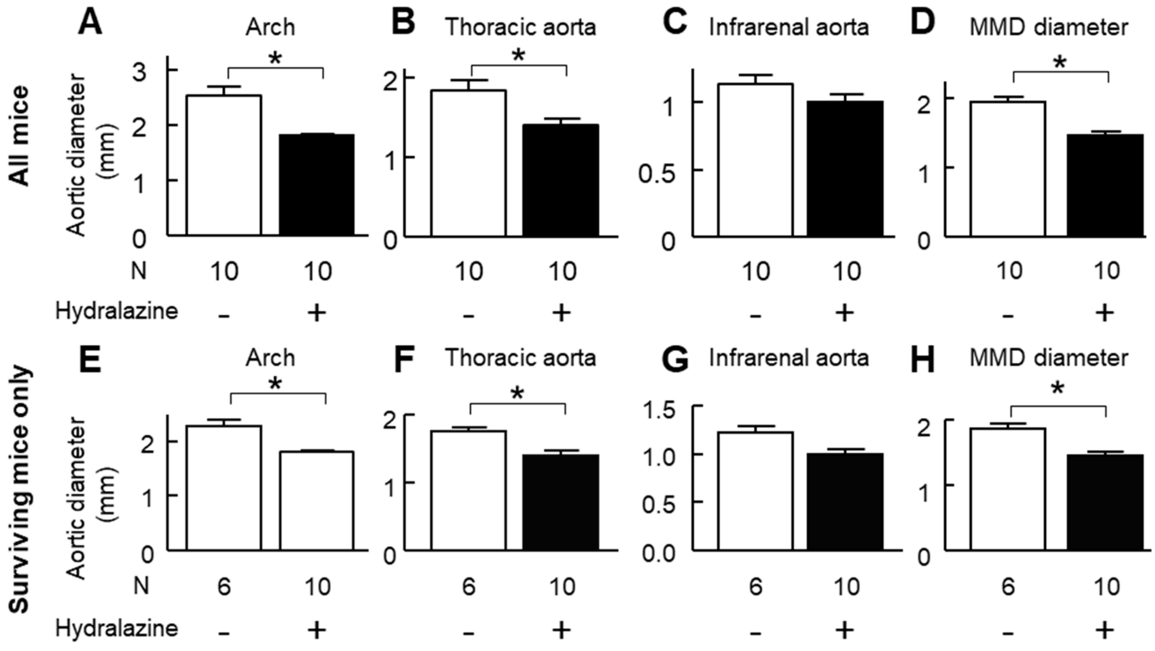


**Figure S3**: Effect of hydralazine treatment on aortic diameters of the aortic arch, thoracic aorta, infrarenal aorta, and mean maximum aortic diameter (MMD). **A-D**, All mice (surviving mice plus dead mice due to aortic rupture). **E-H**, Surviving mice. MMD was the mean of the maximum diameter of the following four aortic segments: aortic arch, descending thoracic aorta, suprarenal aorta, and infrarenal aorta. Error bars = standard error. Data were analyzed using the Mann-Whitney U test. * P<0.05.

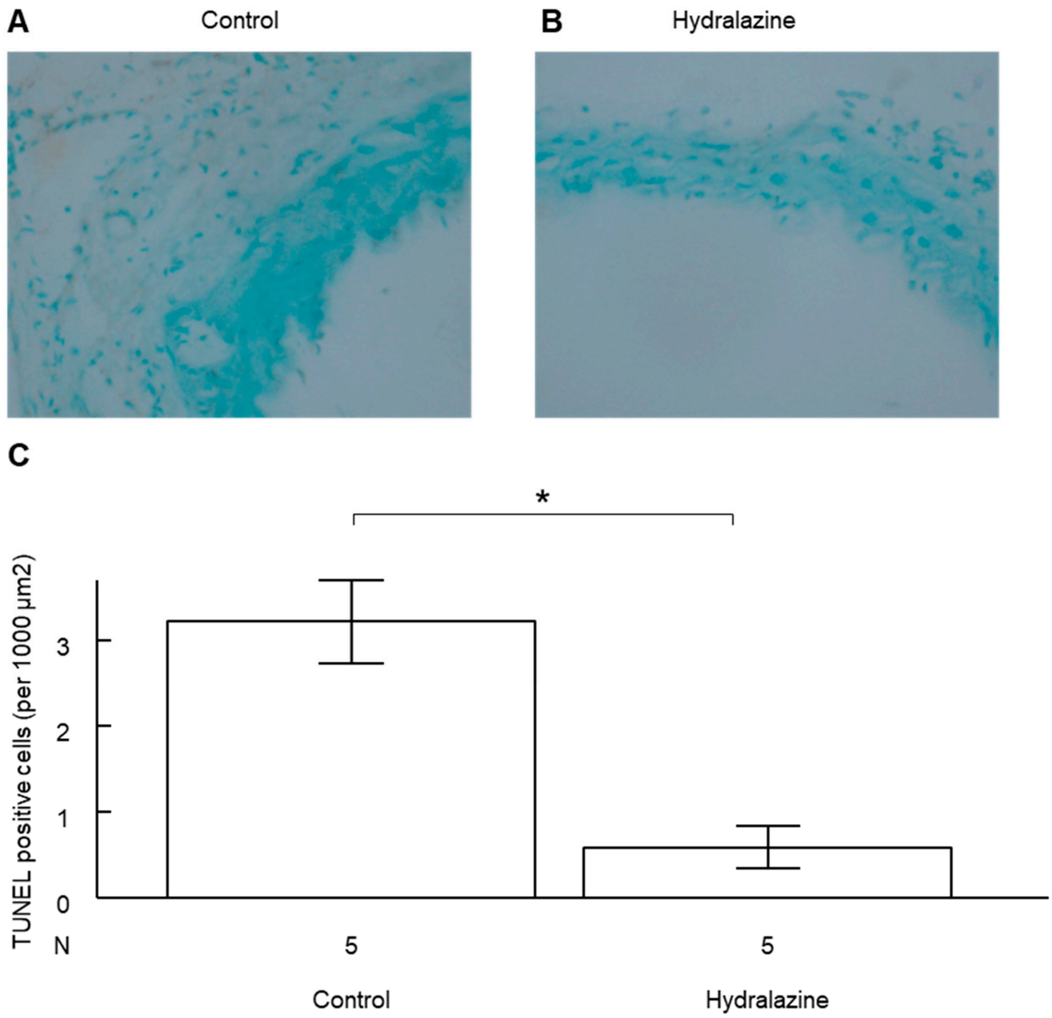


**Figure S4**: Effect of hydralazine treatment on apoptosis. Mice, treated with or without hydralazine (24 mg/kg/day), were sacrificed at the end of the 28-day subcutaneous infusion of angiotensin II (1 μg/kg body/min). The suprarenal aortas were harvested, fixed, sectioned, and stained for apoptosis using the terminal deoxynucleotidyl transferase mediated dUTP nick-end labeling (TUNEL) assay. Nuclei were counterstained with methyl green. **A-B**, Representative images of TUNEL stained aortic sections (magnification, 40X) from the control (untreated, **A**) and hydralazine-treated mice (**B**). **C**, The mean TUNEL-positive cells per 1000 $\mu m^2$. Error bars = standard error. Data were analyzed using the Mann-Whitney U test. * $P<0.05$.

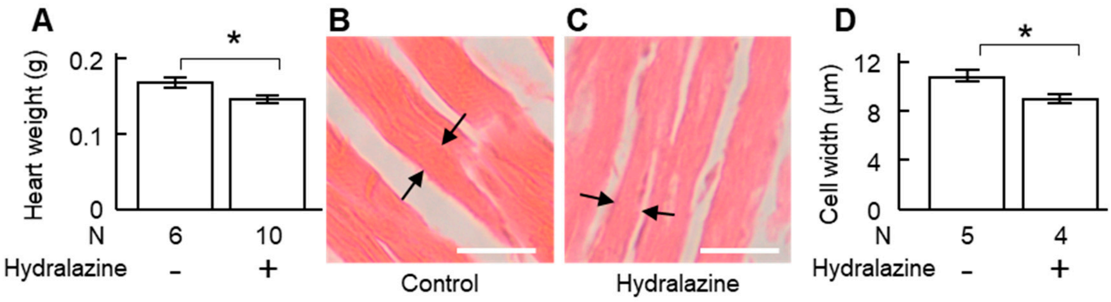


**Figure S5**: Effect of hydralazine treatment on heart weight and cardiomyocyte width. Hearts were harvested from the mice treated with or without hydralazine (24 mg/kg/day) at the end of the 28-day subcutaneous infusion of angiotensin II (1 μg/kg body/min). **A**, Heart weight of the surviving mice. **B-C**, Representative images of cardiomyocyte fibers of the control (untreated, **B**) and hydralazine-treated mice (**C**). Scale bar =20 μM. Between the arrows: cardiomyocyte fibers. **D**, The mean width of cardiomyocytes. Error bars = standard error. Data were analyzed using the Mann-Whitney U test. * P<0.05.
